# Deep learning for medicinal plant species classification and recognition: a systematic review

**DOI:** 10.3389/fpls.2023.1286088

**Published:** 2024-01-05

**Authors:** Adibaru Kiflie Mulugeta, Durga Prasad Sharma, Abebe Haile Mesfin

**Affiliations:** ^1^ Department of Computer Science and Engineering, School of Electrical Engineering and Computing, Adama Science and Technology University, Adama, Ethiopia; ^2^ AMUIT, MOEFDRE under UNDP, MAISM- RTU, Kota, India

**Keywords:** classification, deep learning, medicinal plant dataset, medicinal plant species, recognition

## Abstract

Knowledge of medicinal plant species is necessary to preserve medicinal plants and safeguard biodiversity. The classification and identification of these plants by botanist experts are complex and time-consuming activities. This systematic review’s main objective is to systematically assess the prior research efforts on the applications and usage of deep learning approaches in classifying and recognizing medicinal plant species. Our objective was to pinpoint systematic reviews following the PRISMA guidelines related to the classification and recognition of medicinal plant species through the utilization of deep learning techniques. This review encompassed studies published between January 2018 and December 2022. Initially, we identified 1644 studies through title, keyword, and abstract screening. After applying our eligibility criteria, we selected 31 studies for a thorough and critical review. The main findings of this reviews are (1) the selected studies were carried out in 16 different countries, and India leads in paper contributions with 29%, followed by Indonesia and Sri Lanka. (2) A private dataset has been used in 67.7% of the studies subjected to image augmentation and preprocessing techniques. (3) In 96.7% of the studies, researchers have employed plant leaf organs, with 74% of them utilizing leaf shapes for the classification and recognition of medicinal plant species. (4) Transfer learning with the pre-trained model was used in 83.8% of the studies as a future extraction technique. (5) Convolutional Neural Network (CNN) is used by 64.5% of the paper as a deep learning classifier. (6) The lack of a globally available and public dataset need for medicinal plants indigenous to a specific country and the trustworthiness of the deep learning approach for the classification and recognition of medicinal plants is an observable research gap in this literature review. Therefore, further investigations and collaboration between different stakeholders are required to fulfilling the aforementioned research gaps.

## 1 Introduction

Plants are undeniably valuable sources of medicines, foods, spices, clothing, shelter, fertilizers, and most importantly, elements in climate-change-regulating mechanisms (Abera, 2014). Medicinal plants have played a crucial role in human healthcare for centuries. For instance, Picrorhiza Kurrooa, commonly known as Kutki, has been utilized in traditional medicine to alleviate liver disorders, respiratory issues, and skin conditions. Another example is Swertia Chirayita, which is known for its potential to lower blood sugar levels, protect the liver, prevent cancer, reduce inflammation, and manage fever. Additionally, the Apocynaceae family is believed to offer an alternative treatment option for infections that are resistant to multiple drugs (Pal et al., 2018; Uttpal et al., 2020; Sharma et al., 2021).

Medicinal plants are also widely used in the healthcare systems of the majority of the world’s population. Based on the estimated reports of the World Health Organization (WHO) more than 80% of the developing country’s population uses traditional medicine, whereas herbal medicine has a long history of use for pain relief and disease treatment (Abera, 2014). In addition, medicinal plants show great promise as a source of novel antimicrobial therapies and provide potential opportunities for the development of biocompatible drugs. For example, Withania Somnifera possesses a diverse range of therapeutic properties, such as stress and anxiety reduction, anti-inflammatory effects, immune system modulation, anti-tumor effects, and sexual dysfunction improvement, and has been thoroughly researched for its potential pharmacotherapeutic applications (Anand et al., 2019; Subhabrata et al., 2021).

Plants have been used for medicinal purposes by different civilizations since ancient times until today. Recently, there has been a surge in the use of medicinal plants worldwide, driven by the increasing demand for natural health products, herbal medicines, and secondary metabolites (Chen et al., 2016). For example, Betelvine extracts have demonstrated antimicrobial, antifungal, and antiviral properties, while Piperidine and Piperine exhibit potential anticancer and pharmacological properties (Biswas et al., 2022; Mitra et al., 2022).

According to a conservative estimate, the current loss of plant species is 100 to 1000 times greater than the expected natural extinction rate, and the Earth is losing at least one potential major drug every two years (Bhujun et al., 2017). Worldwide, between 50,000 and 80,000 flowering plant species are used for medicinal purposes, according to the International Union for Conservation of Nature and the World Wildlife Fund (Chen et al., 2016). Approximately 15,000 of these are threatened with extinction due to overharvesting and habitat destruction (Kumar et al., 2021) and with the growing human population and plant consumption, 20% of their wild resources have already been depleted (Chen et al., 2016).

The current extinction rate is largely due to both direct and indirect human activities (Murphy and Romanuk, 2014). So, rapid and accurate medicinal plant species classification and recognition are critical for effective biodiversity research and management. Deep learning, a subfield of machine learning, revolves around training artificial neural networks with multiple layers to autonomously extract data representations (Chanyal et al., 2022). It finds applications in tasks such as the classification of medicinal plant species. Deep learning methods have delivered remarkable outcomes within the field of computer vision, with applications such as image recognition and image enhancement finding widespread adoption across various sectors, including but not limited to healthcare, agriculture, education, and industry (Jiancun et al., 2022; Qiaosen et al., 2022).

In deep learning, there are two primary classification methods: supervised learning, which utilizes labeled data for guidance, and unsupervised learning, where similar patterns are grouped without prior labels (Pushpa et al., 2021). In addressing the challenges within computer vision, researchers are combining both unsupervised deep learning, which leverages unlabeled data, and supervised deep learning, which utilizes labeled data (Pham et al., 2020). Exploiting deep learning to enhance and automate the classification and recognition of medicinal plant species underscores a strong collaborative potential between ongoing botanical research and the application of deep learning techniques.

A recent research review has been conducted to investigate the application of deep learning in the classification of medicinal plants (Chanyal et al., 2022). This article primarily focused on the use of deep learning techniques to classify medicinal plant species. However, for researchers to gain a comprehensive understanding, it is imperative to provide geographical context, specify the dataset employed, identify the most effective deep learning algorithms and techniques, delineate the specific plant components analyzed, highlight the predominantly utilized features, and address areas for potential improvement in the classification and recognition of medicinal plant species. In order to address these issues, we performed a systematic review of the use of a deep learning approach for medicinal plant species classification and recognition issues. In addition the core challenges in deep learning tasks involve dataset management, data preprocessing techniques, and the extraction of relevant features (Guangqi et al., 2022; Lu et al., 2022).

Our systematic review is fundamentally systematized about five key research questions associated with geographical distributions of the research, dataset source, feature extraction, deep learning classifier, and challenges and opportunities for future research. Our predominant interest is to identify best practices and promising research directions for applying deep learning frameworks to medicinal plant species classification and recognition tasks. The notable contributions of this study are summarized as follows:

1. **Publication Demographics: Mapping Global Trends:** Our study quantitatively analyzes the temporal and geographical distribution of publications on medicinal plant species classification and recognition. Investigating beyond statistics, it reveals active research groups and locations, offering nuanced insights into the current frontiers of this dynamic field.
2. **Dataset Dynamics:** This research methodically explores the foundational aspects of datasets vital for training deep-learning models in medicinal plant species classification and recognition. It scrutinizes factors including image quantity, analyzed species, acquisition methods, and preprocessing techniques. The study shows the preparedness of datasets and elucidates strategies, such as image augmentation techniques, employed to enhance dataset quality.
3. **Medicinal Plant Organs and Feature Extraction Techniques:** This study delves into the widespread utilization of medicinal plant organs and their associated feature extraction techniques for the effective classification and recognition of medicinal plant species.
4. **Deep learning methods:** This research provides a comprehensive analysis of state-of-the-art deep-learning methods recommended for tasks in the classification and recognition of medicinal plants. Offering detailed insights into the existing array of deep learning models, it serves as a valuable guide for researchers in the field.
5. **Paving the Way for Future Advancements:** This study systematically investigates the existing challenges and opportunities within the realm of applying deep learning to the classification and recognition of medicinal plant species. Our investigation underscores and highlights fundamental challenges in deep learning tasks for medicinal plant species, with a particular focus on dataset management, data preprocessing techniques, and relevant feature extraction. We also stress the significance of further research in under-resourced countries, where people and animals heavily rely on traditional medicine, reducing geographical distribution barriers.

## 2 Research questions

The studies searched and scrutinized papers using multiple aspects and the review comprises all published research in the field of deep learning methods applied to medicinal plant classifications and recognition and thereby made attempts to answer the following research questions (RQ).

**RQ-1: Demography of publications:** How is the time of publication, location, and research groups distributed across primary studies in medicinal plant species classification and recognition?

**Motivation**: This question aims to retrieve a quantitative overview of the studies, major frontiers, research groups, and locations that are currently active on the topic.

**RQ-2: Dataset preparation and preprocessing techniques:** How many images of how many species of medicinal plants were analyzed per primary study? How were these images acquired? How was the training data preprocessed?

**Motivation**: This question aims to find out how readily the datasets are available for training different deep-learning models for medicinal plant species classification and recognition, to identify the techniques used to generate and preprocess the datasets. Hence, data preprocessing improves data quality, supports feature engineering, and enhances suitability for deep learning, resulting in better model performance and resolution of specific data issues.

**RQ-3**: **Studied organ and feature extraction techniques:** Which parts of the medicinal plant organs are used per primary study? which techniques were used for feature detection and description of medicinal plants?

**Motivation:** this question aims to identify the state-of-the-art organs of the medicinal plant species and feature extraction techniques used in the primary studies for the classification and recognition purpose.

**RQ-4: Deep learning task and method:** Which deep learning method stands out as a top recommendation for the classification and recognition task of medicinal plants?

**Motivation:** This question aims to analyze state of the art deep-learning tasks and methods that have been specifically designed and utilized for the classification of medicinal plant species.

**RQ-5: Challenges and opportunities:** What are the current challenges and opportunities present in the application of deep learning for the classification and recognition of medicinal plant species?

**Motivation**: This question aims to analyze the current challenges that are limiting the application of deep learning and possible research directions that could alleviate these challenges and make these techniques more useful.

## 3 Methodology

### 3.1 Registration and protocol

**Figure 1:**
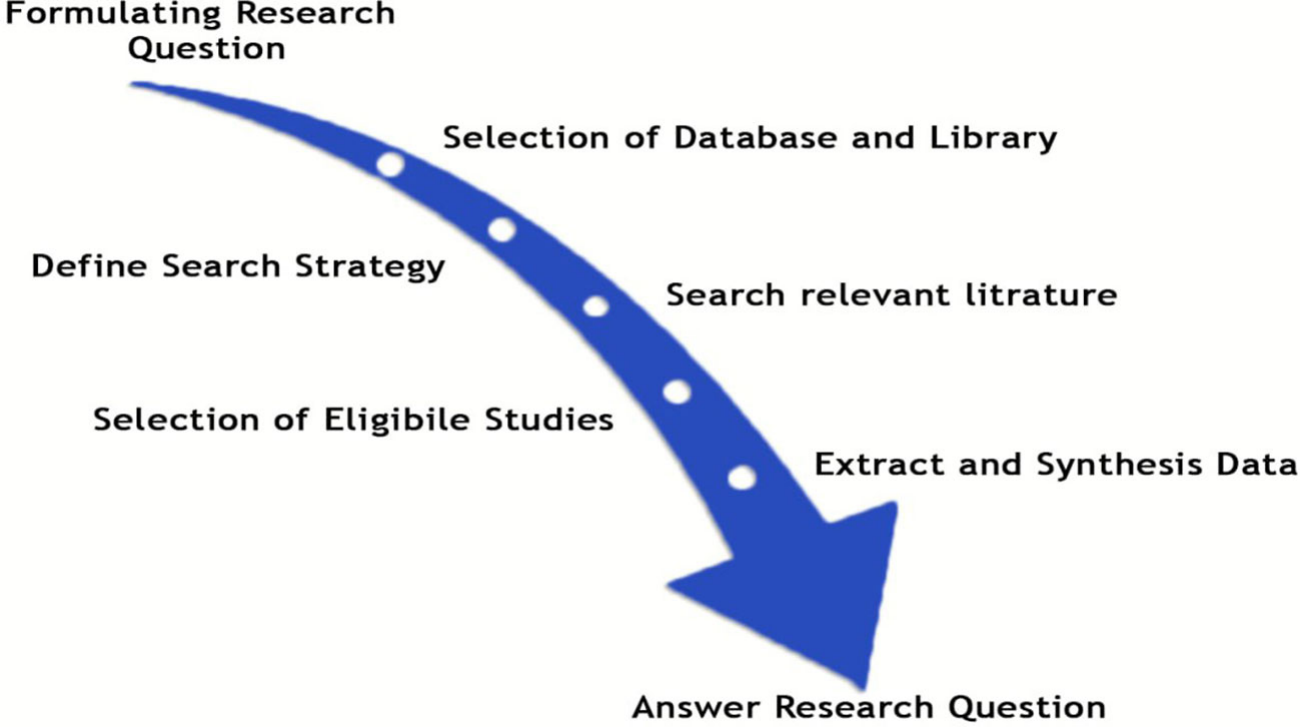
Steps involved in the systematic review protocol.

To present our findings, we adhered to the updated Preferred Reporting Items for Systematic Reviews and Meta-Analyses (PRISMA) guidelines (Antoniou et al., 2021). It’s important to note that we did not register the study protocol in any registry. As depicted in **Figure 1**, we have clearly delineated the comprehensive literature review processes employed in this study.

### 3.2 Eligible criteria

We confined our search to the period between January 2018 and December 2022 to capture the most recent advancements in the classification and recognition of medicinal plants using deep learning. This choice also implies the inclusion of studies from the previous decade. This approach allowed us to focus on emerging trends and gaps in the literature. To identify relevant articles, we assessed whether they met our search criteria based on the paper’s title, keywords, or abstract. Studies that satisfied the specific inclusion and exclusion criteria were included in our review.

The inclusion criteria were determined as:

➢ Research papers published in peer-reviewed journals only (thesis, dissertation, and reports are not included, these papers are not peer-reviewed).
➢ Studies that focus on deep learning for medicinal plant classification or identification.
➢ The research was reported in English.
➢ Containing only full-text article
➢ Studies published between January 2018 and December 2022.

The exclusion criteria were determined as:

➢ Studies that focused on medicinal plant classification and recognition but did not implement deep learning models;
➢ Review studies, abstracts, commentaries, book chapters, conferences, protocols, posters (short papers), or editorials.
➢ Articles that don’t be fully accessed.
➢ Duplicate articles (Using the Endnote reference manager, duplicate articles were removed).

### 3.3 Information source

To find relevant publications in the field, the following five (5) popular databases and libraries were searched: Science Direct, Google Scholar, PubMed, IEEE Xplore, and SpringerLink. This ensured that we only focused on high-quality reputed publications. We searched these terms in titles, keywords, and abstracts.

### 3.4 Search strategies

Prior to initiating the systematic study, it is vital to choose the most effective search query to maximize the retrieval of relevant articles that can address the research question. After multiple trial-and-error iterations, the ultimate search query is as follows: [“deep learning” OR “convolutional neural network” OR “recurrent neural network” OR “computer vision” OR “artificial neural network” OR “artificial intelligence”) AND (“indigenous medicinal plant” OR “medicinal plant” OR “traditional medicinal plant” OR “ayurvedic plant” OR “herbarium plant” OR “ethnomedicinal plant”) AND (“identification” OR “recognition” OR “detection” OR “classification”)] were used to obtain published articles.

### 3.5 Selection process

After completing the search process, we combined all retrieved entries from the five search engines into a single comma-separated value (CSV) file, and any duplicate entries were removed. Subsequently, two researchers independently evaluated the titles and abstracts of all non-duplicate records based on the eligibility criteria. In cases of disagreement, a third researcher acted as a mediator. Following this initial screening, two researchers worked together to thoroughly examine the full texts of the previously selected records to determine their eventual inclusion in the review.

### 3.6 Data collection process

We created a data extraction form designed for gathering information from the chosen papers. This form was initially used by one of our review authors and subsequently cross-validated by another author. Any discrepancies or disagreements were openly discussed and collectively resolved.

### 3.7 Study selection

**Figure 2:**
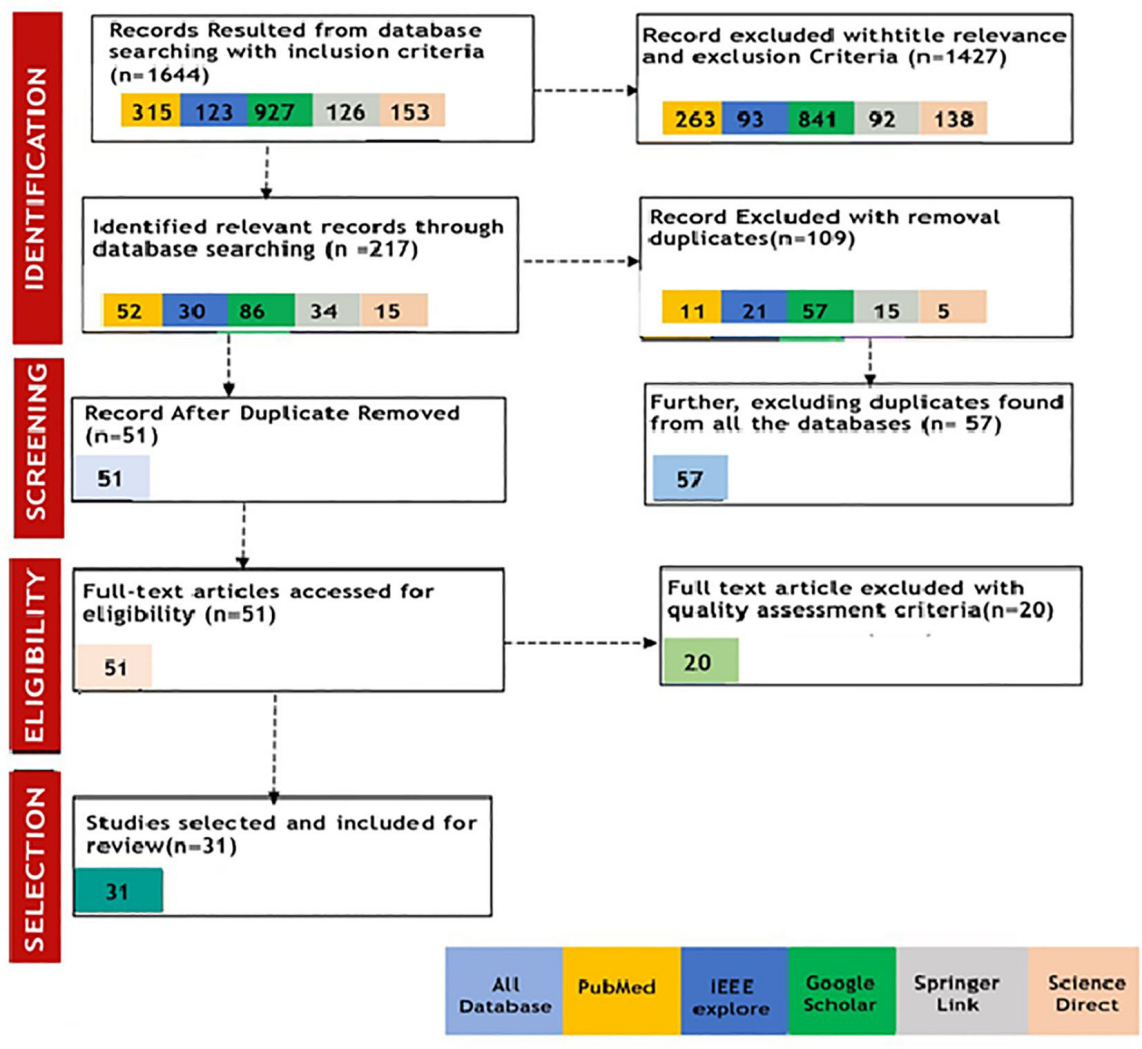
The PRISMA framework for research screening process.

**Figure 2** illustrates the complete PRISMA 2020 framework, depicting our selection process following the eligibility criteria. Through the implementation of our search strategies and selection criteria, we successfully located a total of 1644 studies across all the mentioned databases. To break this down further, we collected 927 article records from Google Scholar, 315 from PubMed, 126 from Springer Link, 153 from Science Direct, and 123 from IEEE Explore, all based on our designated search keywords. By applying the PRISMA methodology for screening research papers, we carefully refined the selection process, resulting in the identification of 31 studies that satisfied the inclusion criteria for our systematic review.

### 3.8 Data extraction

In this systematic review, we carefully analyzed all primary studies and extracted the necessary data. To collect the required information for the research questions (RQ) RQ-1, RQ-2, RQ-3, and RQ-4 in a structured manner, data extraction templates (**Table 1**) are designed. To answer RQ-1, corresponding information was extracted mostly from the meta-data of the primary studies. The others are mostly analyzed from the methodology and other related parts of the primary studies. Nevertheless, in addressing RQ-5, no specific template was developed; instead, the focus is on assessing gaps and potential future avenues based on the primary studies.

**Table 1:** Data extraction review template.

Demography of publication	Dataset preparation and preprocessing techniques
Study Year of publication [2018–2022] Country of all author(s) Authors’ Background	No of Species No of images Image Source: [Private, Public] Image Type [photo, scan] Name of Dataset Data augmentation techniques Image processing techniques
**Studied organ and feature extraction techniques**	**Deep learning tasks and methods**
Studied organ[leaf, flower, fruit, stem] Studied features Extraction techniques	Deep Learning Method Deep Learning Task

### 3.9 Quality assessment

To ensure the quality of the reviews, it is critical to assess the threat of validity. Quality assessment and validity parameters were also considered in this literature review. The selection and assessment of the primary studies were based on the designed eligibility criteria above, then the criteria we used were defined so that the studies could be assessed based on their clarity, aim, scope and context. We used publication titles, abstracts, and keywords to search for our primary studies. We have made a comprehensive effort to design eligibility criteria that capture a broad range of relevant literature. Consequently, our search has incorporated a substantial number of studies that fulfill our criteria. The final results remained objective-leveled though. The same applies to data extraction and grouping data after data extraction so that they could be handled much easier. At this point, it is worth mentioning that there were primary studies in which data were not mentioned at all, or might have been missed or misinterpreted. That stands on the fact that the data extraction is objective too. For example, there might be ‘medicinal plant species identification’ in the Title and Abstract of the publication, fulfilling the selection criteria, but the Text of the specific publication was not mentioned as an identification task but only as a segmentation task instead.

It was observed that the threat of validity arises from two aspects which were the bias in study selection and the bias in the data extraction process. Study selection depends on the search strategy used. To mitigate this bias, we performed both manual and automatic searches across multiple databases and our search strategy was very inclusive. Furthermore, we established a data extraction template that was diligently adhered to in order to address the research questions.

### 3.10 Data analysis and synthesis

To identify the most commonly used and currently popular deep learning models for classifications and recognitions of medicinal plant species, we began our critical review by formulating a set of research questions. The primary studies that were pertinent to the research questions were then extracted using an empirically developed search string. Later we used a set of predetermined inclusion and exclusion criteria to classify the pertinent pieces of research works. After selecting a handful of pertinent research papers, we extracted the needed data to respond to the research questions from the selected research papers.

After a rigorous analysis of these studies, we described the geographical distribution and locations of the primary research effort and then found out how readily the datasets are available for training the different deep-learning models for the classification tasks of medicinal plant species. We described which medicinal plant organs are essential for the specified tasks, and analyzed the current deep-learning classifiers, with the main performance metrics used by the primary studies. Finally, the paper points out the current challenges and opportunities for classifying medicinal plant species. This analysis produced a future research roadmap that outlines the theoretical underpinnings of deep learning methods utilized in research on medicinal plant species classifications.

### 3.11 Basic terminologies

Image classification assigns a single class to the entire image, while object detection recognizes multiple objects, determining their locations with bounding boxes. Segmentation explores the detailed spatial distribution, delineating boundaries and identifying pixels for specific objects (Xu et al., 2023a).. Recognition is a comprehensive term for identifying patterns, objects, or entities (Cheng et al., 2018). Deep leering task is a class of machine learning techniques employing artificial neural networks with multiple layers. These sophisticated networks autonomously learn intricate patterns and relationships from vast datasets, excelling in diverse tasks such as image recognition, classification, and complex problem-solving, revolutionizing various fields through their predictive capabilities (Yu, 2022; Xu et al., 2023a).

Data augmentation techniques enriches datasets by applying transformations like rotations, flips, and color adjustments (Xu et al., 2023b). Image processing techniques manipulate images for tasks such as enhancement, restoration, and feature extraction, contributing to an overall improvement in data quality (Nixon and Aguado, 2019). A photo (leaf photo) captures a live plant’s leaf in its natural habitat, providing a visual representation of the leaf’s characteristics in its original environment. In contrast, a scanned picture represents a preserved herbarium specimen (Goëau et al., 2021).

## 4 Results

### 4.1 Demography of publication

**Figure 3:**
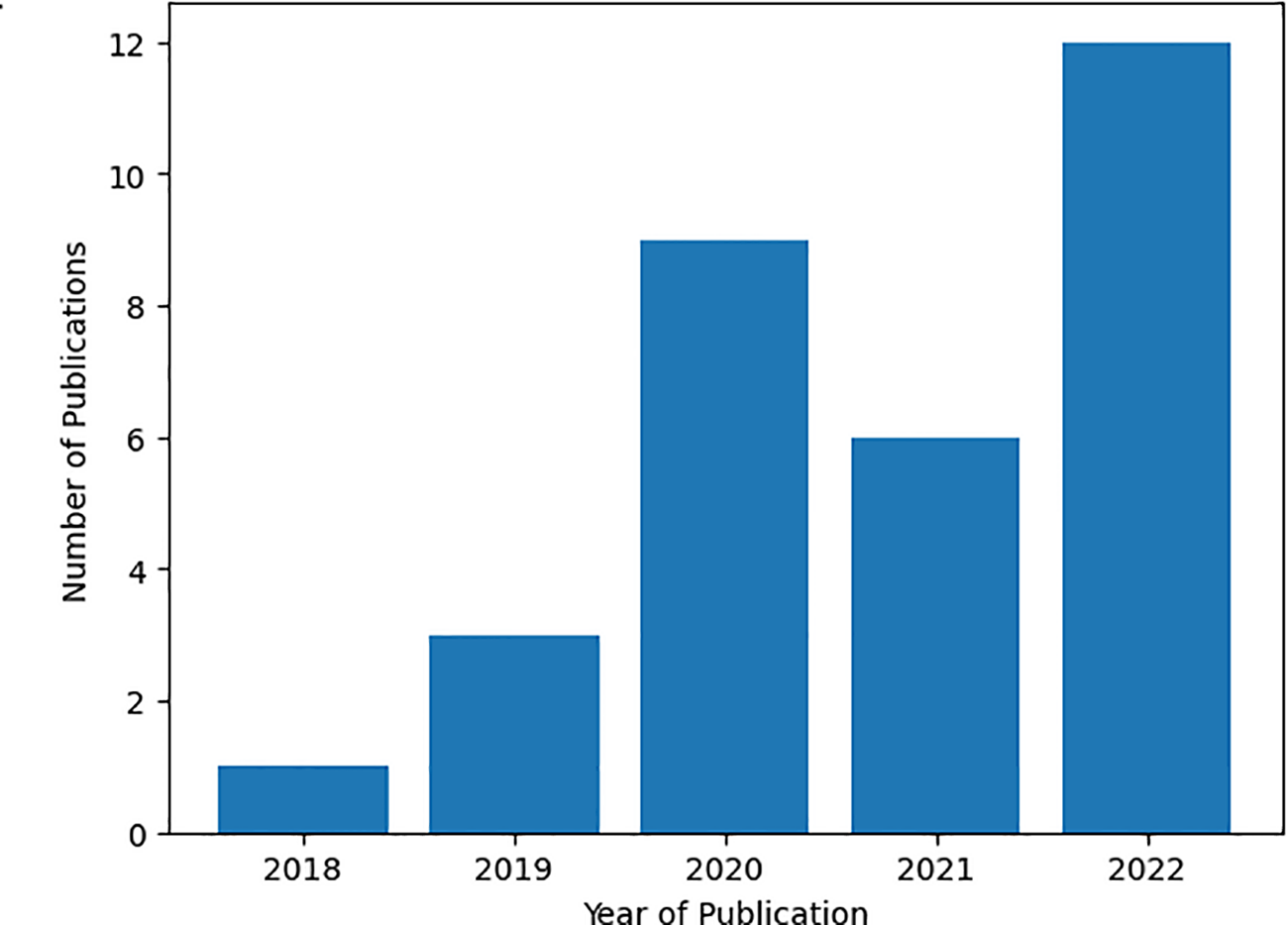
Number of studies per year of publication.

We conducted an analysis where we aggregated research paper statistics based on their publication years to investigate the evolving interest in medicinal plant classification and recognition, as depicted in **Figure 3** below. The data clearly illustrates a notable upward trend in the number of publications from 2018 to 2022. These findings point to a growing and sustained interest in this particular research domain, as demonstrated by the consistent increase in the quantity of published papers in recent years. To illustrate, in 2022, approximately 12 papers were published, while only one was produced in 2018. This observation strongly suggests that researchers currently view the research topic as highly significant and worthy of exploration.

**Figure 4:**
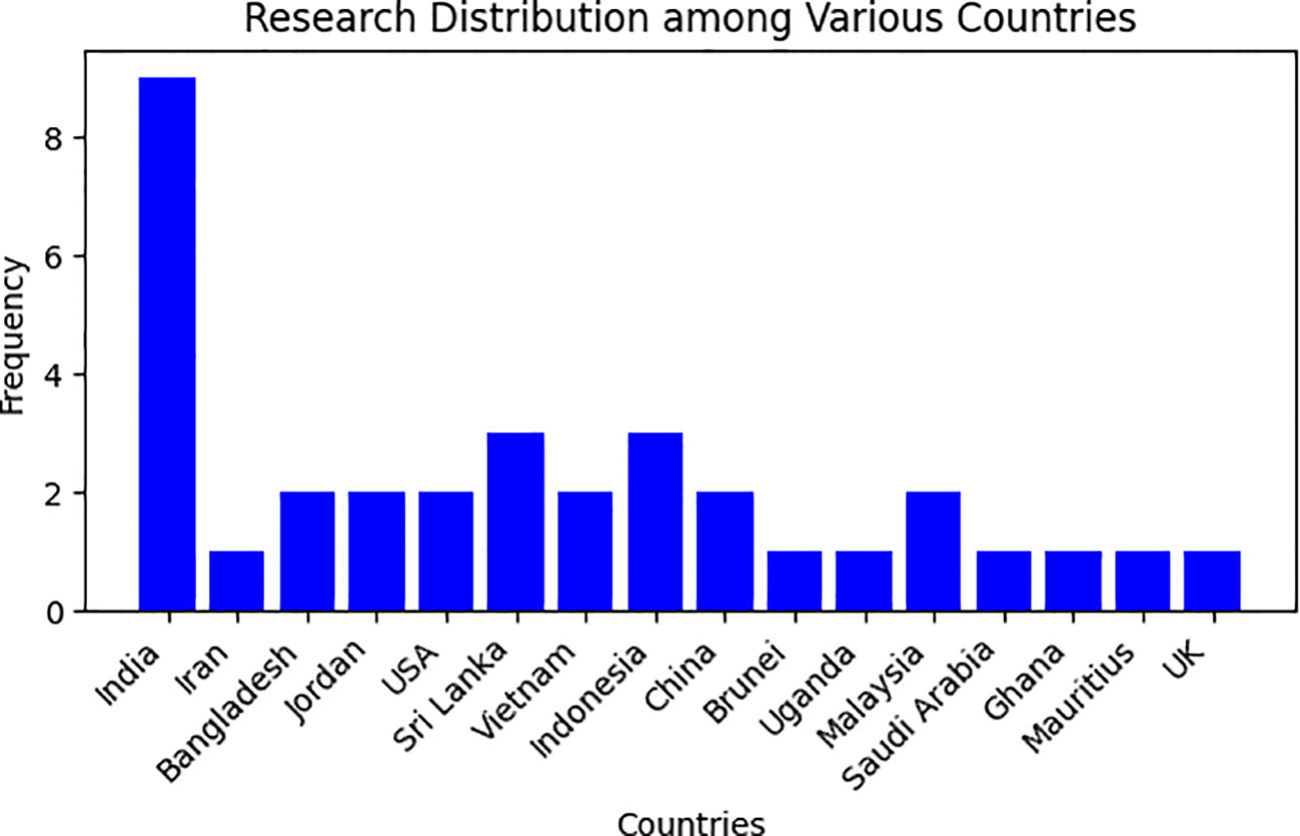
Distribution of research across different Countries.

To gain a comprehensive understanding of active research groups and their geographical distribution, we conducted an analysis of the affiliations of the first authors. This information has been visually represented in **Figure 4**.

**Table 2** presents our findings, which indicate that the selected papers were authored by researchers from 16 different countries. The majority of papers 29% (n=9) had a first author from India, followed by Indonesia and Sri Lanka, which accounted for 19% (n=6) of the papers each. Additionally, Bangladesh, China, Vietnam, and the USA together contributed 25.8% (n=8) of the papers. Almost all of the papers analyzed in the study were written entirely by researchers with backgrounds in deep learning fields (computer science, information systems, information technology, or computer science and engineering). As evident from the **Figure 4**, only a select number of countries are actively engaged in the identification and classification of medicinal plants. Consequently, there is a pressing need for a robust initiative to stimulate further research efforts in this field. This urgency arises from the study’s alarming revelation that medicinal plants are currently at risk of endangerment.

**Table 2:** Geographic regions of origin.

Study Identifier	Publication Year	Countries of the Author	Author’s Background
(Liu et al., 2018)	2018	China	Deep learning field
(Anubha Pearline et al., 2019)	2019	India	Deep learning field
(Azeez and Rajapakse, 2019)		Sri Lanka	Deep learning filed
(Islam et al., 2019)		Bangladesh	Deep learning field
(P and Patil, 2020; Paulson and Ravishankar, 2020)	2020	India	Deep learning field
(Akter and Hosen, 2020)		Bangladesh	deep learning field
(Indrani et al., 2020)		Indonesia	Deep learning field
(Jayanka and Fernando, 2020; Senevirathne et al., 2020)		Sri Lanka	Deep learning field
(Little et al., 2020)		USA	Agriculture field
(Muneer and Fati, 2020)		Malaysia and Saudi Arabia	Deep Learning field
(Quoc and Hoang, 2020)		Vietnam	Deep learning field
(Dat et al., 2021)	2021	Vietnam	Deep learning field
(Diqi and Mulyani, 2021)		Indonesia	Deep learning field
(Huang et al., 2021)		China	Deep learning field
(Joshi et al., 2021; Roopashree and Anitha, 2021)		India	Deep learning field
(Pudaruth et al., 2021)		Mauritius	Deep learning and Agriculture field
(Abdollahi, 2022)	2022	Iran	Deep learning field
		Jordan, USA	Deep learning field
		India	Deep learning field
(Haryono et al., 2020)		Indonesia	Deep learning field
(Malik et al., 2022)		Brunei, Uganda	Deep learning field
(Oppong et al., 2022)		Gahanna	Deep learning field
(Pushpanathan et al., 2022)		Malaysia	Deep learning and Agriculture field
(Shahmiri et al., 2022)		UK	Deep learning field

### 4.2 Dataset preparation and preprocessing

This comprehensive review extensively explores datasets specific to Medicinal Plant Species, forming the foundation for deep learning methods in plant classification and recognition tasks. It intricately dissects data acquisition methods, origin sources, image volume per species, data extraction protocols, data augmentation techniques, and image typologies tailored for task-specific needs. Additionally, the review underscores the challenges inherent in dataset preparation and preprocessing within the context of medicinal plant classification. The detailed insights presented in **Table 3** serve as a valuable reference. Through this comprehensive analysis, the goal is to enhance comprehension of dataset utilization within the realm of deep learning-enabled medicinal plant classification and recognition.

**Table 3:** Dataset, data augmentation techniques, and data preprocessing techniques.

Study	Dataset	Source Type	No of Images	No of Species	Image Type	Augmentation Techniques	Preprocessing Techniques
(Akter and Hosen, 2020)	Bangladeshi Medicinal Plant	Private	37,693	10	Photo	Flip and Rotation	Normalization and Scaling
(Huang et al., 2021)	Blossom	Private	12,538	12	Photo	Filter, Brighter, Darker, Rotation, Noise Increase	Delete Blurred Images, Cropping, Position Adjustment
(Almazaydeh et al., 2022)	Mendeley	Public	1,800	30	Scan	Resize	Segmentation
(Divyasree and Sheelarani, 2022)			1,835	30	Photo	–	Resizing and Augmentation
(Sachar and Kumar, 2022)			1,841	30	Photo	–	–
(P and Patil, 2020)	Not Available	Private	7,150	7	Photo	Random Rotation, Flip and Random Noise	Image Segmentation, Morphological change
(Azeez and Rajapakse, 2019)			512	7	Photo	–	–
(Haryono et al., 2020)			4,050	9	Photo	–	Resizing
(Indrani et al., 2020)			5,454	9	Photo	–	Bitmap and conversion
(Islam et al., 2019)			8,000	8	Photo	–	Resizing and Orientation
(Jayanka and Fernando, 2020)			2,180	17	Scan and Photo	Rotation and Mirroring	Background Removal and Resize
(Paulson and Ravishankar, 2020)			64,000	64	Photo	–	–
(Pudaruth et al., 2021)			7,000	70	Photo	–	Resize
(Senevirathne et al., 2020)			1,500	6	Photo	–	Morphological process
(Tiwari et al., 2022)			4,502	12	Photo	–	–
(Liu et al., 2018)	Chines Herbal Medicine	Private	8,500	50	Photo	–	Resize
(Roopashree and Anitha, 2021)	DeepHerb	Private	2,515	40	Photo	Flip, Rotation, Scaling, and Addition of Noise	Gaussian blur, Edge Detection, salt, and pepper noise removal
(Diqi and Mulyani, 2021)	Face94	Private	500	5	Photo	–	Thresholding, erosion and dilation
(Dat et al., 2021)	Flavia, Leafsnap, Folio-Herbs	Private and Public	10,325	268	Photo	Rotation	Gaussian filter
(Anubha Pearline et al., 2019)	Folio, Swidish leaf, Flavia,leaf12	Private and Public	7,511	91	Photo	–	Gaussian filter
(Little et al., 2020)	Herbarium-2019	Private and Public	46,469	683	Photo and Scan	–	Gaussian Filter, PhotoOCR and Resize
(Banita Pukhrambam and Sahayadhas, 2022)	IMPPAT	Private and Public	48,632	1,742	Photo	–	Adaptive Vector Median Filter and an Average Detection Filter
(Al-Qurran et al., 2022)	Herbarium 2020 FGVC7 plants	Public	1,000,000	32,000	Scan	Rotation, Flip, Zoom Range,	–
(Joshi et al., 2021)	Inleaf	Private	4,500	10	Photo	Mirroring, Rotation and Cropping, Zooming and Changing Color Saturation	Manual Labeling and Bias Removal using Data Augmentation
(Muneer and Fati, 2020)	Malaysian Herbs	Private	1,000	20	Scan	–	Canny Edge Detection, Adaptive Threshold and grayscale conversion
(Abdollahi, 2022)	Medicinal-Plants	Private	3,000	30	Photo	Rotation and Gaussian blur	Normalization and Cropping
(Oppong et al., 2022)	MyDataset	Private	2,450	49	Photo	–	Log Gabor and Features Extractor
(Pushpanathan et al., 2022)	MYLPHerbs-1	Private	34,200	12	Scan	–	–
(Malik et al., 2022)	UBD Botanical dataset, PlantClef	Private and Public	115,302	106	Photo and Scan	Merging	–
(Quoc and Hoang, 2020)	VNPlant-200	Private	20,000	200	Photo	Rotate, Zoom and Resize	–
(Shahmiri et al., 2022)	VNPlant-200	Public	20,000	200	Photo	–	Mutual Information Guided Training (MIGT) Algorithm

**Figure 5:**
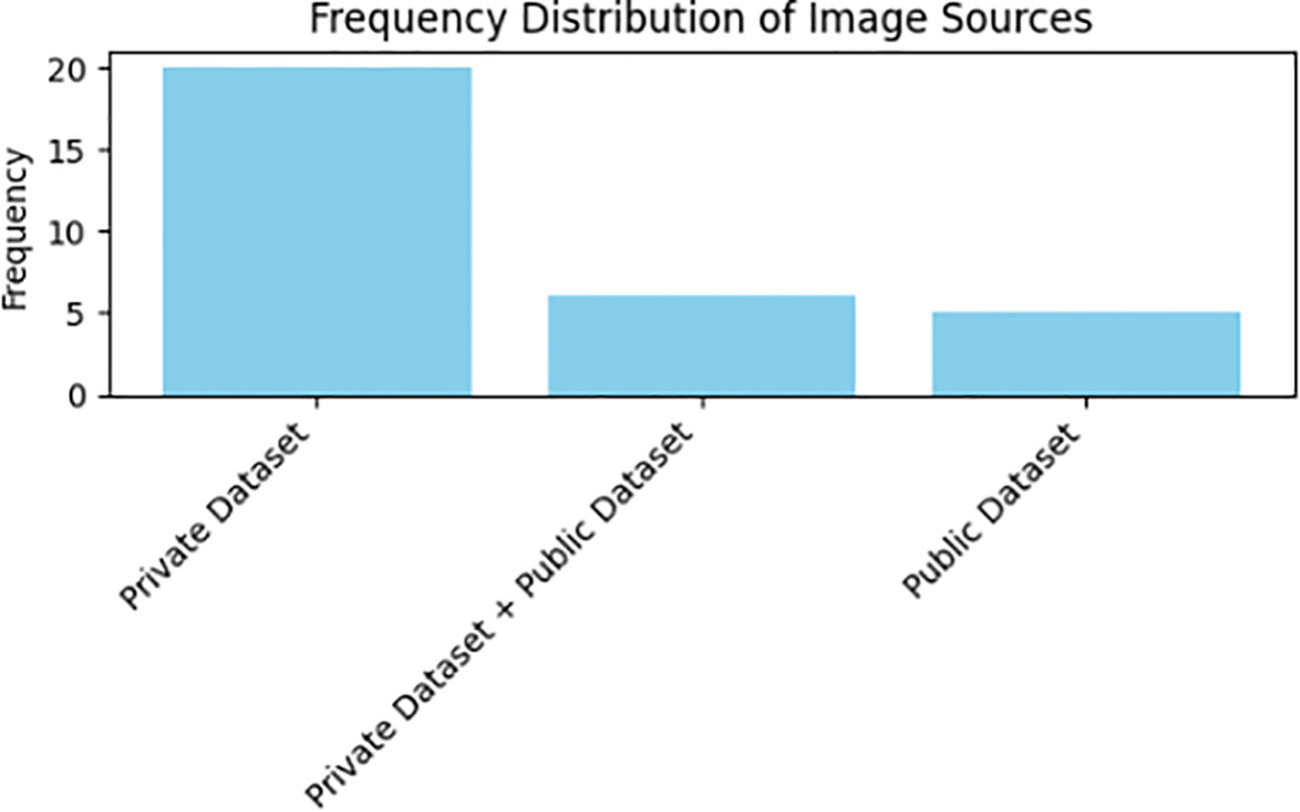
Distribution of data source types.

As demonstrated in **Table 3**, primary studies consistently lean toward utilizing private datasets in their pursuit of employing deep learning techniques for the classification and recognition of Medicinal Plant Species. This observation is substantiated by insights presented in **Figure 5**. The review’s findings underscore that a noteworthy 67.8% (n=21) of primary studies opted for their private exclusive datasets. Additionally, 16.1% (n=5) of the studies selected public datasets tailored to similar challenges, while an equivalent percentage of 16.1% (n=5) amalgamated their private datasets with public dataset.

In our analysis, as depicted in **Figure 5**, a prominent trend emerged in the primary studies regarding the usage of images for the classification and recognition of medicinal plant species, as outlined in **Table 3**. Specifically, a notable 54.8% (n=17) of primary studies utilized an extensive number of images exceeding 5000, whereas 45.1% (n=14) employed fewer than 5000 images. Furthermore, a significant majority of researchers, constituting 77.4% (n=24) of the studies, collected data on medicinal plant species that exceeded 10. Only a minority, comprising 22.6% (n=7) of researchers, opted for a smaller number of medicinal plant species for deep learning tasks within the primary studies. Notably, from the results of these primary studies, a substantial 74.1% (n=23) employed image preprocessing techniques, while 38.7% (n=12) employed data augmentation methods on the raw dataset.

### 4.3 Studied organs and feature extraction techniques

Deep feature extraction in deep learning involves capturing intricate and abstract representations through neural networks. It’s the procedure of compressing data into meaningful attributes for analysis, like reducing an image to key features such as shape or colors (Nixon and Aguado, 2019). Feature extraction techniques, like CNNs for images or RNNs for sequential data, enable automatic extraction of high-level features, facilitating nuanced learning representations in complex tasks (Nixon and Aguado, 2019). For the classification and recognition processes of medicinal plant images, feature extraction is essential after the preprocessing steps. The main aims of the feature extraction in the classification and recognition processes are dimensionality of reeducation the information by extracting characteristics patterns from the leaf image of the medicinal plant. **Table 4** shows the studied organs, and feature extraction techniques of the reviewed primary studies.

**Table 4:** Studied organs, and feature extraction techniques.

Study	Studied Organ	Feature Extraction Techniques
(P and Patil, 2020)	Leaf	Digital Morphological Features (DMF) and VGG16
(Abdollahi, 2022)		Morphological, Texture, and Shape Feature Extraction
(Pushpanathan et al., 2022)		VGG16, VGG19, ResNet50, EfficientNet B0 and EfficientNet B7
(Tiwari et al., 2022)		AlexNet, VGG-19, ResNet-101, and DenseNet201
(Muneer and Fati, 2020)		Zernike, Hu(shape) and Texture (Gray-Level Co-Occurrence Matrices(GLCM))
(Dat et al., 2021)		UNet
(Oppong et al., 2022)		AlexNet,Inceptionv3,DenseNet201,GoogleNet, Resnet101, Resnet50, Mobilenetv2, VGG16 and VGG19
(Senevirathne et al., 2020)	Leaf and Flower	FRCNN(Faster Recurrent Convolutional Neural Network)
(Akter and Hosen, 2020)	Leaf	Attention-based feature map
(Almazaydeh et al., 2022)		Resnet101 with FPN(Feature Pyramid Network)
(Al-Qurran et al., 2022)		InceptionV3, ResNetV2 and InceptionResNetV2.
(Anubha Pearline et al., 2019)		VGG16, VGG19, InceptionV3 andInception-ResNetV2
(Azeez and Rajapakse, 2019)		InceptionV3, ResNet, MobileNet and InceptionResNetV2
(Banita Pukhrambam and Sahayadhas, 2022)		DenseNet
(Diqi and Mulyani, 2021)		Susuki Algorithm
(Divyasree and Sheelarani, 2022)		VGG16
(Haryono et al., 2020)		CNN-LSTM
(Huang et al., 2021)		AlexNet, InceptionsV3
(Indrani et al., 2020)		SqueezeNet
(Islam et al., 2019)		YOLOV2
(Jayanka and Fernando, 2020)		Multi-layer perceptron (MLP), CNN
(Joshi et al., 2021)		ResNet50
(Little et al., 2020)		SeResNeXt-101, ResNet-50,SeNet-154
(Malik et al., 2022)		EfficientNet-B2
(Paulson and Ravishankar, 2020)		VGG16 and VGG19
(Pudaruth et al., 2021)		Inception-v3
(Quoc and Hoang, 2020)		VGG16, VGG19, Resnet50, InceptionV3, Densenet121, Xception and MobileNetV2
(Roopashree and Anitha, 2021)		VGG16, VGG19, InceptionV3 and Xception
(Sachar and Kumar, 2022)		MobileNetV2, InceptionV3, and ResNet50
(Shahmiri et al., 2022)		VGG16, VGG19, Resnet50, InceptionV3, Densenet121, Xception and MobileNetV2
(Liu et al., 2018)		GoogleNet

According to the data presented in **Table 4**, an examination of medicinal plant organs, the features employed for classification and recognition, and the extraction techniques reveals a prominent pattern within primary studies. Notably, an overwhelming majority of 96.8% (n=30) of these studies focused on utilizing leaf organs for these purposes, with only one study opting for a combination of leaves and flowers. In terms of feature extraction, most primary studies 83.8% (n=26) used a pre-trained model with transfer learning. Only a small percentage of studies 16.1% (n=5) used alternative techniques such as digital morphological changes (P and Patil, 2020), morphological changes (Abdollahi, 2022), attention-based feature map (Akter and Hosen, 2020), Susuki Algorithm (Diqi and Mulyani, 2021), and Zernik, Hu for shape extraction, as well as GLCM for texture extraction (Muneer and Fati, 2020).

### 4.4 Deep learning task and method

A deep learning classifier, employing neural networks, categorizes data by learning hierarchical features from extensive datasets. In contrast, deep learning tasks have distinct goals, such as image classification (Sharifi et al., 2022). In deep learning approach, features are autonomously extracted directly from RGB images as input, allowing the model to extract meaningful features for classification purpose. This is distinct from traditional machine learning, which relies on image processing methods to obtain color, texture, and shape indexes as part of feature extraction. In the realm of scientific computing, deep learning has garnered significant popularity, finding application in businesses addressing intricate challenges. Notably, Convolutional Neural Networks (CNN), Deep Neural Networks (DNN), and pretrained models are prevalent classification techniques. **Table 5** presents a visualization of deep learning classifiers, associated tasks, and their respective performance metrics.

**Table 5:** Deep learning task, method, and performance.

Study	DL Task	DL Method	Performance
(P and Patil, 2020)	Classification	CNN	Accuracy =98%
(Abdollahi, 2022)		MobileNetV2	Accuracy=98.3%
(Akter and Hosen, 2020)		CNN	Accuracy=71.3% Precision= 66.4% Recall 67.6% F1-Score=66.4%
(Almazaydeh et al., 2022)		Mask-RCNN	Accuracy=98.7%
(Al-Qurran et al., 2022)		End-To-End CNN	–
(Anubha Pearline et al., 2019)		VGG19 and Logistic Regression	Folio Dataset Precision=97% Recall=97% F1-Score=97% Accuracy=96.53% Swedish Leaf Precision=96% Recall=96% F1-Score=96% Accuracy=96.25% Flavia Precision=96% Recall=96% F1-Score=96% Accuracy=96.25% Leaf12 Precision=97% Recall=97% F1-Score=97% Accuracy=97.14%
(Azeez and Rajapakse, 2019)		PNN	Accuracy= 71.4%
(Diqi and Mulyani, 2021)		CNN	Precision=22% F1-score=23% Recall=22.5% Accuracy=86%
(Divyasree and Sheelarani, 2022)		CNN	Accuracy 99%
(Huang et al., 2021)		CNN	Precision=98.4% Recall=98.5% F1-Score=98.45% Accuracy =93.53%
(Indrani et al., 2020)		CNN	Accuracy=81%
(Islam et al., 2019)		CNN	Accuracy 96%
(Jayanka and Fernando, 2020)		MLP	Accuracy=58.7% Accuracy=97.71%
		CNN	
(Joshi et al., 2021)		CNN	Accuracy=99.43%
(Little et al., 2020)		SeResNext-101	Accuracy =89.8%
		ResNet-50	Accuracy=89.8
		SeNet-154	Accuracy 89%
(Malik et al., 2022)		CNN	Accuracy 82.6%
(Muneer and Fati, 2020)		DNN	Accuracy=93%
(Oppong et al., 2022)		OTAMNet(hybrid of DenseNet201 and Log Gabor)	Accuracy =97%
(Paulson and Ravishankar, 2020)		VGG16	Accuracy=97.8% Accuracy=97.6%
		VGG19	
		CNN	Accuracy=95%
(Pudaruth et al., 2021)		VGG16	Accuracy =90%
(Pushpanathan et al., 2022)		VGG 19	Accuracy=90%
(Quoc and Hoang, 2020)		Xception Xception	Accuracy=88.26%
(Roopashree and Anitha, 2021)			Accuracy=97.5%
(Sachar and Kumar, 2022)		Ensemble	Accuracy=99.4%
(Shahmiri et al., 2022)		CNN	Accuracy=97%
(Liu et al., 2018)		CNN	Accuracy=89.4%
(Haryono et al., 2020)		CNN	Accuracy=94.6%
(Senevirathne et al., 2020)		FRCNN	Accuracy=98%
(Tiwari et al., 2022)		CNN	Accuracy=98.7%
(Banita Pukhrambam and Sahayadhas, 2022)	Classification and Segmentation	CNN-DenseNet	Sensitivity =99.25% Specificity= 99.56% Accuracy= 99.78% F1-scor=61%
(Dat et al., 2021)	Segmentation	Multimodal- CNN	Accuracy=98.9% Avg. Computational Cost =0.220

**Figure 6:**
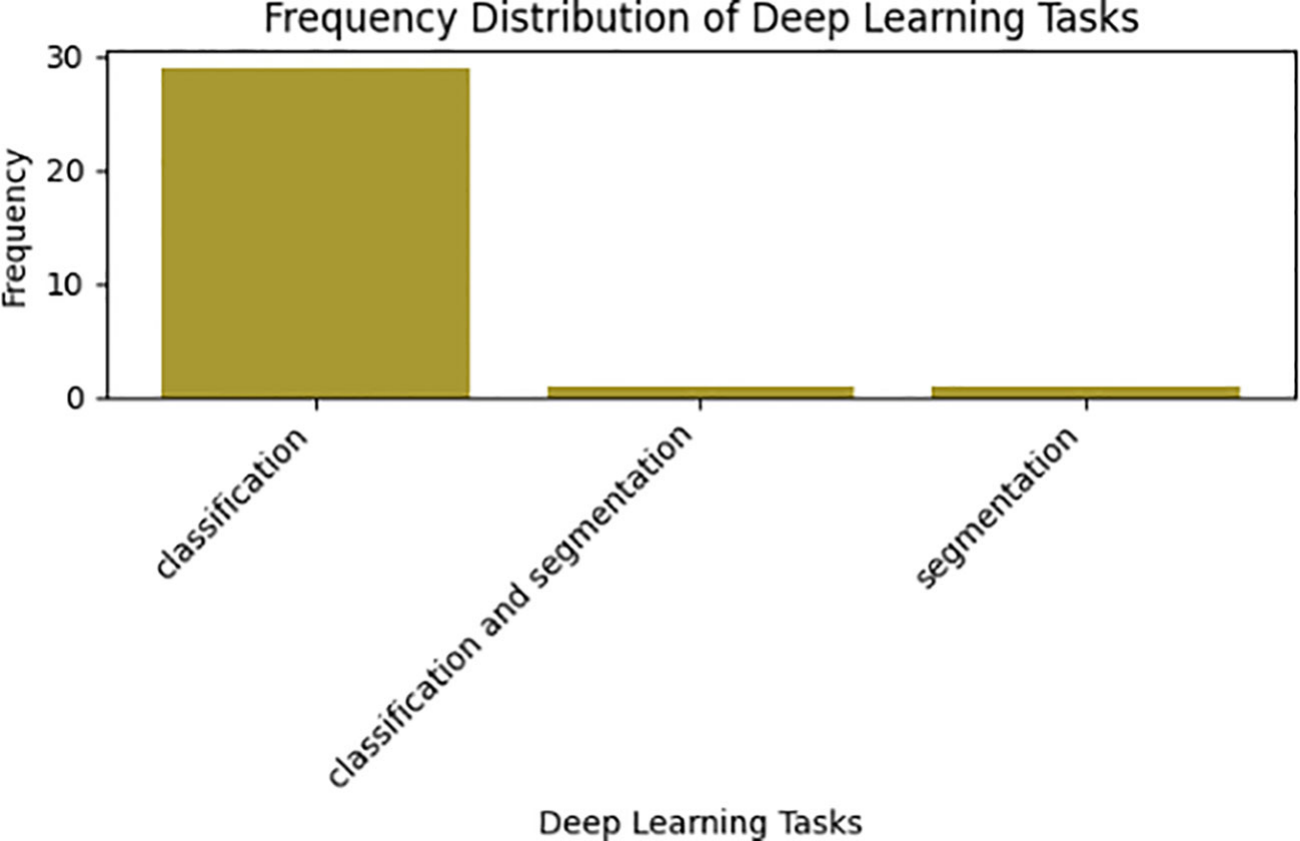
Distribution of deep learning tasks.

**Table 5** provides a comprehensive visual representation that underscores the prevalence of deep learning techniques across a spectrum of tasks within medicinal plant research, encompassing classification, identification, segmentation, and their combined applications. Upon meticulous analysis of the reviewed data, a clear trend emerges: a significant majority of primary studies favored the use of deep learning for classification and recognition tasks. Hence, all of the primary studies assessed in this studies employed supervised deep learning techniques to address the identification and classification challenges of medicinal plant species, as visually depicted in **Figure 6**. Specifically, 93.5% (n=29) of these studies opted for classification techniques. Additionally, certain studies explored alternative avenues, including segmentation, and the fusion of classification and segmentation methodologies. Furthermore, the Convolutional Neural Network (CNN) emerged as the predominant deep learning method employed in medicinal plant tasks. Among the examined studies, 64.5% (n=20) utilized CNN, while others incorporated distinct methods such as hybrid approaches like CNN-DenseNet (Banita Pukhrambam and Sahayadhas, 2022), along with traditional machine learning algorithms Logistic Regression (LR). Additionally, studies employed DNN (Muneer and Fati, 2020), end-to-end CNN (Al-Qurran et al., 2022), MobileNetV2 (Abdollahi, 2022), Mask-RCNN (Almazaydeh et al., 2022), PNN (Azeez and Rajapakse, 2019), and Xception (Quoc and Hoang, 2020; Roopashree and Anitha, 2021).

## 5 Discussion

### 5.1 Demography of publications

Our research findings enthusiastically highlight an increasing interest in the field. The results also reveal a pronounced concentration of studies originating from Asian countries, with Indian researchers notably taking the lead. This revelation underlines the importance of addressing a significant research gap in other regions, where there might be variations in research sophistication. Importantly, it’s worth noting that African countries are currently underrepresented in this landscape, despite their abundant medicinal plant resources. For instance, consider Ethiopia, where the reliance on traditional medicines by around 80% of the population is culturally resonant and cost-effective (Aragaw et al., 2020). However, it’s paradoxical that Ethiopia’s diverse indigenous medicinal plants face vulnerability due to factors like a scarcity of experts and limited awareness. This reality emphasizes the urgency of engaging communities for the identification, monitoring, conservation, and sustainable use of these vital resources. Consequently, the adoption of deep learning-based approaches for medicinal plant species classification and recognition emerges as a pertinent solution to address these pressing challenges.

The analysis outcome additionally indicates that a majority of academic studies related to the classification and recognition of medicinal plant species are predominantly authored by individuals from the computing fields. However, an interdisciplinary collaboration among people from different fields and backgrounds is necessary to achieve effective collaborative research activities and develop widely accepted approaches (Kyvik and Reymert, 2017). This is also applicable to the classification and recognition of medicinal plant species. For instance, in the paper (Chen and Sun, 2018) discusses the importance of interdisciplinary collaboration between botanists, medicinal plant researchers, and other professionals to promote conservation, education, and research on medicinal plants. This underscores the necessity for interdisciplinary collaborations to enhance insights into medicinal plant species. Such partnerships are pivotal for addressing the challenges of identification and classification, paving the way for solving related issues in the future.

### 5.2 Dataset preparation and preprocessing

In this study, we analyzed the quantity of images and medicinal plant species in primary research studies. We pinpointed preprocessing methods for these images and aimed to identify the specific plant organ segments used for these tasks. Additionally, we sought to differentiate the techniques used in creating and improving these datasets.

Our findings highlighted that custom datasets are the primary approach within primary studies concentrating on deep learning methods for the classification and recognition of medicinal plant species. Alongside this, open-source datasets like Flavia, Swedish leaf, and Folio also gained popularity. Notably, in studies involving indigenous medicinal plants, the creation of a custom dataset proves vital in capturing the nuanced aspects of plant diversity, cultural context, traditional knowledge, research objectives, and ethical considerations, especially intertwined with indigenous communities (Heinrich et al., 2009). Furthermore, a consistent trend emerged where photographic images of medicinal plants obtained from natural surroundings were preferred by a majority of primary studies, retaining the inherent environmental context. We observed a positive correlation between the augmentation of image quantities or datasets and improved performance in deep learning tasks. However, the absence of standardized datasets and preparation protocols poses a notable challenge in this field. We also examined the use of image augmentation techniques and the mechanisms behind the processing of the medicinal image datasets. Deep learning models are not amenable to raw data for the tasks of deep learning rather the dataset is subjected to preprocessing and formatting before being given to the designed deep learning model. Data preprocessing and augmentation techniques can enhance the accuracy and robustness of deep learning models, reduce overfitting, and improve generalization performance, which is crucial for designing accurate and reliable deep learning models for the identification and classification of Medicinal Plant Species. Augmentation techniques enhances training data diversity, thus preventing overfitting and improving model generalization (Deng et al., 2009).

The preparation and preprocessing of datasets bear immense significance in deep learning tasks. These practices enhance data quality, reduce redundancy, and prime the data for analysis, ultimately bolstering the overall performance of deep learning models (Deng et al., 2009; Fan et al., 2021). Standard data processing typically involves data cleaning, normalization, and augmentation (Deng et al., 2009; Fan et al., 2021). Data cleaning encompasses the removal of duplicate, low-quality, and inaccurately labeled images, ensuring high-quality data and consequently elevating model performance. Notably, normalization plays a critical role in dataset preparation, scaling pixel values to a standardized range, leading to improved model convergence and accuracy.

In the realm of dataset composition, it’s imperative to conduct thorough assessments. This includes examining image sources and accompanying metadata, like plant species information. Sample inspections involving the random selection of images from diverse categories aid in understanding the range and variability of medicinal plant species. Maintaining dataset quality involves manual inspection of a subset of labeled images to ensure accuracy and consistency. Addressing potential data imbalances entails implementing label validation and counting labels to rectify underrepresented classes. It’s crucial to assess how data imbalances impact model performance and prediction accuracy. Techniques like oversampling, under sampling, and transfer learning with pre-trained models are valuable for balancing class distribution, particularly in cases with limited data.

### 5.3 Studied organs and feature extraction

Recognizing one or more characteristics of a medicinal plant and linking them with a name, either a common or so-called scientific name, is required for species identification. Experts typically use the plant size, shape as a whole, flowers color, flower size, flower growing position, flower inflorescence, plant stem shape, plant stem node, plant stem outer character, plant stem bark pattern, plant fruits size, fruits color, fruits quality, and plant leaves shape, leaves margin, leaves pattern, leaves texture, and leaves vein (Wäldchen and Mäder, 2018). The basic characteristic of medicinal plant patterns is found in texture, shapes, and colors (Gonzalez, 2009; Wäldchen and Mäder, 2018). This study highlights that among primary studies, leaf shape analysis received the most attention for classifying and identifying medicinal plants. Leaf shape is preferred over leaf geometry because it is considered more heritable and less affected by environmental factors. Despite the detailed differences between the leaves of different Medicinal Plant Species, these differences are often apparent to botanists. Furthermore, because leaves can be easily distinguished from plain backgrounds, they are identified as the easiest feature to automatically extract for the classification and recognition process. Although transfer learning using pre-trained models is the most commonly used technique for extracting features from medicinal plant images, the selection of organs and features, along with their descriptions, remains the most challenging aspect of the process.

### 5.4 Deep learning tasks and methods

Analyzing the efficiency of deep learning across a wide range of tasks involving medicinal plants and identifying the dominant deep learning classifier algorithms employed for these tasks poses an intricate difficulty. An analysis of primary research reveals that deep learning methods, encompassing families like VGG16 (Paulson and Ravishankar, 2020; Pudaruth et al., 2021), VGG19 (Paulson and Ravishankar, 2020; Pushpanathan et al., 2022), CNN (Akter and Hosen, 2020; Indrani et al., 2020; P and Patil, 2020; Paulson and Ravishankar, 2020), MobileNetV2 (Abdollahi, 2022), DenseNet (Banita Pukhrambam and Sahayadhas, 2022; Oppong et al., 2022), Faster-RCNN (Senevirathne et al., 2020) and Xception (Quoc and Hoang, 2020; Roopashree and Anitha, 2021), have attained accuracy levels surpassing 97% for tasks linked to categorizing, recognizing, and segmenting Medicinal Plant Species.

This finding indicates the superior performance of deep learning models compared to traditional machine learning classifiers, underscoring their significant advantages in this particular field. Indeed, Convolutional neural networks (CNNs) have gained widespread usage in image classification tasks due to their strong capability to analyze visual data and automatically learn significant features from images (Wiestler and Menze, 2020; Abdollahi, 2022; Pathak et al., 2022; Bharadiya, 2023).

Deep learning has the ability to automatically learn relevant features from raw data, eliminating the need for manual feature extraction and selection (Annavarapu, 2021; Pathak et al., 2022). These models can effectively scale and handle large datasets, which is particularly advantageous in domains with extensive labeled datasets (Wiestler and Menze, 2020; Pathak et al., 2022). Pretrained models using transfer learning approach leverages the potentials of solving the issues of dataset shortage in various domain. Transfer learning has been employed by leveraging pre-trained models, such as those trained on large-scale datasets like ImageNet, and fine-tuning them for specific tasks (Shin et al., 2016; Cui et al., 2018; Pathak et al., 2022). This approach helps overcome limitations caused by limited medicinal plant datasets. By using a pre-trained model as a feature extractor, the model can learn high-level features from images and then fine-tune them with a smaller medicinal plant dataset for classification.

Instead of training a deep neural network from scratch, transfer learning allows us to fine-tune the pretrained model on a smaller dataset related to the target task. VGG16, VGG19, Inception families, ResNet Families, and DenseNet Families are frequently used pre-trained models for the classification and recognition of medicinal plant species.

To address challenges arising from limited datasets and uncertainties, while also enhancing model generalization and performance, it is recommended to utilize fusion methods. These methods involve the integration of information from various data sources to achieve these objectives. Ensemble methods have also been used to improve the overall performance of deep-learning classifiers (Elhariri et al., 2014; Annavarapu, 2021; Chompookham and Surinta, 2021; Sachar and Kumar, 2022). These methods combine multiple classifiers and aggregate predictions to reduce errors and enhance accuracy.

### 5.5 Current deep learning approaches: challenges and opportunities

In the discussion section of this systematic review, it is evident that the majority of researchers collected and designed custom datasets for medicinal plant species within their study areas or countries. Consequently, these solutions cannot be directly applied. This emphasizes the significance of feature-based intelligent customization and contextualization to attain greater precision and accuracy in the classification of medicinal plant species. Additionally, a contextualized customization of existing solutions with different datasets may reveal new knowledge for the classification and recognition of medicinal plant species that are endemic to a specific country. The lack of available medicinal plant species datasets represents a research gap in this area. Furthermore, it is apparent that different deep learning approaches have limited scope to experimentally explore and address the challenges and issues related to classifying and identifying medicinal plant species. This is because deep learning approaches provide classification and recognition without sufficient explanation and are considered untrustworthy due to their black-box nature.

While significant progresses have been made in the realm of deep learning, numerous challenges persist and uncharted opportunities remain within current approaches. It is imperative to tackle these challenges and seize the opportunities to ensure the continuous success and advancement of deep learning. One potential benefit of using deep learning methods for data analysis is the possibility of achieving higher accuracy and automation in solving classification, identification, and recognition problems. deep learning also provides an opportunity to handle large amounts of data efficiently (Deng et al., 2009; Fan et al., 2021). Transfer learning is a technique that enables pre-trained models to be used as a starting point for new tasks, reducing the amount of training data required and improving performance (Taylor and Stone, 2009; Shin et al., 2016; Dong et al., 2019; Zhuang et al., 2021). Developing deep learning models that are interpretable and can explain their predictions is an essential area of research, particularly in fields such as medicinal plants, where model decisions can have significant consequences (Yan et al., 2020; Vinuesa and Sirmacek, 2021; Li et al., 2022).

However, deep learning faces several challenges, including the need for high-quality labeled data, model interpretability, and a lack of standardized protocols for dataset preparation, data preprocessing, and analysis. Deep learning models require a massive amount of labeled data to achieve high accuracy, and collecting and labeling large datasets can be challenging and time-consuming. Model interpretability is difficult, as deep learning models are often considered “black boxes.” Overfitting is another challenge, as deep learning models can easily memorize training data instead of learning generalizable patterns, leading to poor performance on unseen data (Justus et al., 2018; Rudin, 2019; Gastounioti and Kontos, 2020; Rasley et al., 2020). Additionally, training deep learning models can be computationally expensive and require specialized hardware such as GPUs, posing challenges for real-time data analysis (Justus et al., 2018; Rasley et al., 2020).

Hence, it is imperative to tackle these challenges through the development of more resilient and interpretable deep learning models, establishing standardized protocols for dataset preparation and preprocessing, and optimizing computational resources for the real-time analysis of classification, identification, and recognition issues.

## 6 Conclusion

Classifying medicinal plant species from digital images is a difficult task. The deep learning approach using CNN architecture has proved to be capable of adequately dealing with the most challenges associated with medicinal plant species classification and recognition. This review paper concentrates mainly on a state-of-the-art review of deep learning models used in the field of medicinal plant species classification and recognition. In this study, we presented a systematic review of the primary studies related to deep learning from the domain of medicinal plant classification and recognition. The study assessed the deep learning approach used to classify and identify medicinal plant species with the consideration of the geographical distribution of the paper, the availability of medicinal plant dataset sources, the techniques used for pre-processing the dataset, the feature extraction techniques used, the deep learning classifier used, and the performance metrics used for measurement.

The majority of the papers are from India, then followed by Indonesia and Sri Lanka. A private dataset with pre-processing and augmentation techniques was used by the majority of researchers. As a feature extractor, transfer learning with a pre-trained model was used, and CNN was used as a deep learning classifier.

The review can help the researchers understand how a deep learning approach can be used to classify and identify Medicinal Plant Species. The challenging issues, limitations, and feature open areas of research related to the field were also discussed in detail.

In the future, it is crucial for researchers to prioritize addressing significant research gaps. These gaps are notably characterized by the scarcity of datasets and geographical disparities, which underscore the lack of research in developing countries. Despite deep learning’s impressive performance in classifying and identifying medicinal plant species, trustworthiness remains a key concern. To address this issue, there is a pressing need to develop interpretable deep learning models. This endeavor should also encompass the establishment of standard protocols for dataset management and the creation of high-quality labeled datasets. Furthermore, researchers should focus their efforts on creating country specific datasets, taking into account the indigenous nature of many medicinal plants. Additionally, optimize computational resources to effectively address real-time challenges in classification of medicinal plant species. A clear understanding in these domains equips researchers with the necessary tools for effective utilization of deep learning models in medicinal plant identification and classification. Our research is crucial for traditional medicine and pharmaceutical advancements, improving recognition and supporting conservation goals.

## Author contributions

AKM: Conceptualization, Data curation, Formal analysis, Funding acquisition, Investigation, Methodology, Project administration, Resources, Software, Supervision, Validation, Visualization, Writing – original draft, Writing – review & editing. DS: Methodology, Resources, Supervision, Validation, Visualization, Writing – review & editing. AHM: Data curation, Formal analysis, Software, Writing – review & editing.
